# Visualizing Strain‐Coupled Cryogenic Phase Transitions and Defect Dynamics in Perovskite Quantum Dots Using In Situ STEM

**DOI:** 10.1002/advs.202516496

**Published:** 2025-12-07

**Authors:** Xinjuan Li, Zhao Jiang, Si Chen, Yi Tang, Bofeng Xue, Tianhao Wu, Yang Lu, Xavier Moya, Akshay Rao, Zhongzheng Yu, Caterina Ducati

**Affiliations:** ^1^ Department of Materials Science and Metallurgy University of Cambridge Cambridge CB3 0FS UK; ^2^ Cavendish Laboratory University of Cambridge JJ Thomson Avenue Cambridge CB3 0HE UK

**Keywords:** cryogenic phase transition, defect healing dynamics, in situ electron microscopy, perovskite quantum dots, strain localization

## Abstract

Perovskite quantum dots (PeQDs) offer high photoluminescence quantum efficiencies, precise spectral tunability, and solution‐processability, making them promising for advanced optoelectronics. However, their structural and defect evolution under thermal stress remains poorly understood. Here, direct nanoscale insights are provided into temperature‐driven phase transition and defect dynamics in CsPbBr_3_ PeQDs using high‐resolution, high‐angle annular dark‐field scanning transmission electron microscopy (HAADF‐STEM) images, 4D STEM, and photoluminescence spectroscopy. Sub‐ångström imaging at room temperature reveals inherent atomic features and octahedral tilting of the lead halide perovskite lattice in PeQDs, suggesting a pre‐tilted, low‐symmetry state before thermal perturbation. The cryogenic cooling induces a reversible orthorhombic‐to‐monoclinic phase transition, distinct from bulk perovskite behavior and accompanied by severe strain localization exceeding 20% at surfaces and grain boundaries. A controlled cryogenic post‐synthesis treatment can effectively heal defects and improve radiative recombination, whereas prolonged cryo‐treatment introduces irreversible structural degradation. These findings highlight the intrinsic structural flexibility of PeQDs and provide a scalable post‐synthesis treatment method to optimize the stability and efficiency of QDs for various optoelectronic applications.

## Graphical abstract

Cryogenic cooling induces an orthorhombic‐to‐monoclinic phase transition in CsPbBr_3_ quantum dots, accompanied by pronounced strain localization at surfaces and interfaces. Multimodal in situ STEM directly visualize reversible defect healing during moderate cryogenic treatment and irreversible degradation upon prolonged exposure, revealing the intrinsic coupling between structural dynamics, strain evolution, and optical performance in halide perovskites.

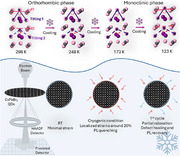

## 1 Introduction

The rapid advancement of optoelectronics has driven significant interest in lead halide perovskite quantum dots (PeQDs), due to their superior photophysical properties.^[^ ^1^, ^2^, ^3^, ^4^, ^5^ ^]^ These materials exhibit high photoluminescence quantum efficiencies (PLQEs), precisely tunable emission peaks, and excellent processability, making them promising candidates for next‐generation single‐photon emitters, light‐emitting diodes (LEDs), lasers, solar cells and photodetectors.^[^ ^6^, ^7^, ^8^, ^9^, ^10^, ^11^ ^]^ However, the stability and operational longevity of PeQDs remain major challenges, particularly under thermal cycling and low‐temperature conditions. Among various types of PeQDs, CsPbBr_3_ QDs stand out for their superior performance, including excellent optical performance, structural and spectral stability, and well‐characterized phase transitions, making them a model system for probing nanoscale perovskite behavior.^[^ ^7^, ^12^ ^]^

Temperature variations can profoundly influence the structural integrity and optoelectronic properties of PeQDs.^[^ ^13^, ^14^, ^15^, ^16^ ^]^ These materials exhibit unique phase transition behaviors and defect dynamics at the nanoscale that significantly deviate from their bulk counterparts due to the quantum confinement effect, surface chemistry, and ligand interactions. While bulk CsPbBr_3_ primarily adopts an orthorhombic phase at room temperature, recent studies suggest that nanoscale size effects may introduce additional structural instabilities, phase transitions, and lattice distortions.^[^ ^1^, ^4^ ^]^ Furthermore, defects such as halide vacancies and surface traps play a crucial role in non‐radiative recombination, directly impacting device efficiency and long‐term reliability.^[^ ^17^, ^18^ ^]^ High‐temperature investigations have revealed reversible lattice expansion and octahedral tilting toward cubic symmetry, accompanied by red‐shifted emission and photoluminescence quenching driven by enhanced electron–phonon coupling.^[^ ^19^, ^20^ ^]^ These processes delineate the upper bound of the thermodynamic phase response. However, the corresponding low‐temperature regime remains largely unexplored, even though cooling provides the inverse pathway of these transformations, driving lattice contraction, symmetry lowering, and strain localization that can fundamentally alter defect states and carrier dynamics. Therefore, a comprehensive understanding of these temperature‐induced transformations across the full thermal range is critical for optimizing PeQDs’ structural and optoelectronic stability.

Several previous studies have investigated perovskite structural evolution, phase transitions, and defect healing strategies. X‐ray diffraction (XRD) and Raman spectroscopy have provided insights into bulk phase behavior, while transmission electron microscopy (TEM) and scanning probe techniques have been employed to study surface defect distributions.^[^ ^21^, ^22^, ^23^, ^24^ ^]^ However, these conventional methods often lack the spatial resolution or sensitivity to capture atomic‐scale distortions and local strain variations within individual PeQD. In particular, the precise structural rearrangements and phase transformations occurring under cryogenic conditions remain largely unexplored and challenging.

To address these gaps, we employ a multimodal approach integrating advanced atomic‐resolution imaging, temperature‐dependent 4D scanning transmission electron microscopy (4D‐STEM) with photoluminescence (PL) spectroscopy. This combination enables direct visualization of how structural transformations, strain evolution, and defect dynamics at the nanoscale affect optical properties, providing unprecedented insights into the fundamental mechanisms that dictate PeQD stability and performance. We focus on elucidating how PeQDs structurally evolve under cryo‐cycling to uncover nanoscale mechanisms of defect healing and degradation. Finally, we investigate whether our controlled cryogenic post‐synthesis treatment can improve the optical performance of PeQDs.

Our findings reveal a previously unreported orthorhombic‐to‐monoclinic phase transition in CsPbBr_3_ QDs under cryogenic conditions, accompanied by strain redistribution and atomic‐scale distortions. Atomic‐resolution HAADF‐STEM imaging at room temperature captures nanoscale octahedral features and structural disorder, while simulations show octahedral tilting and spacing changes upon cryogenic cooling. We further demonstrate that cryogenic post‐synthesis treatment (1–2 h) effectively relaxes strain and enhances radiative recombination. In contrast, prolonged cryo‐exposure leads to detrimental structural distortions. These findings establish a novel temperature‐driven defect engineering strategy to enhance the performance of PeQDs. By bridging nanoscale structural insights with macroscopic optical behavior, this work offers a foundational framework for advancing the PeQDs‐based optoelectronics.

## 2 Results and Discussion

### 2.1 Structural Characterization of CsPbBr_3_ PeQDs at Room Temperature

**Figure 1:**
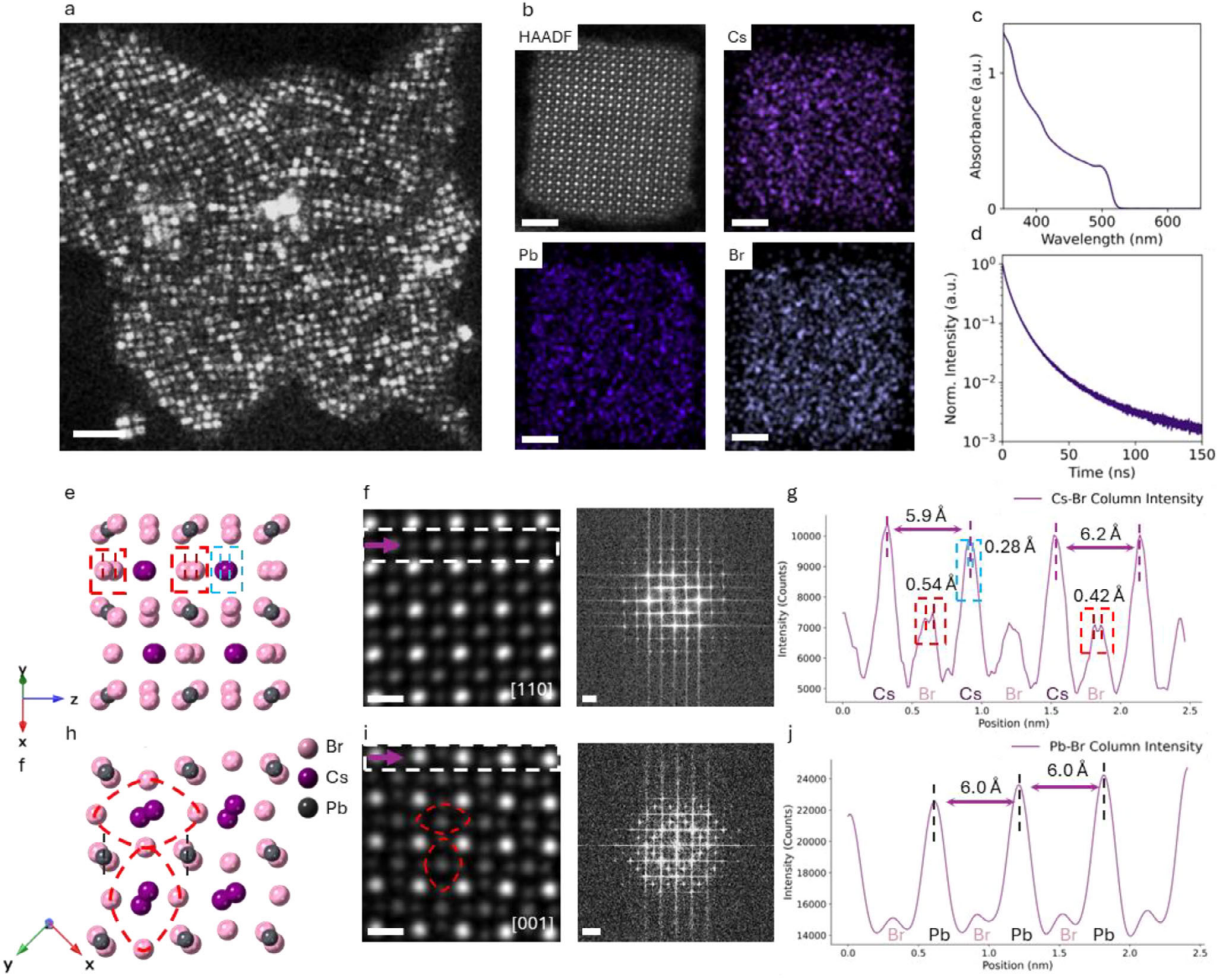
Structural, optical, and compositional characterization of CsPbBr_3_ QDs. a) Virtual dark field (vDF) image reconstructed from 4D‐STEM data, revealing the spatial distribution and size of CsPbBr_3_ QDs. The median size is 6.5 nm. Scale bar, 50 nm. b) STEM‐EDX elemental mapping of a single QD confirming the homogeneous distribution of Cs, Pb, and Br, consistent with the expected stoichiometry. Scale bar, 2 nm. c) PL spectrum of pristine CsPbBr_3_ QDs, exhibiting a sharp emission peak at 514 nm, characteristic of the green‐emitting perovskite phase. d) TCSPC lifetime measurement, indicating a PL lifetime of 9.1 ns. e) Simulated structure of CsPbBr_3_ along the [100] axis. f) Atomic‐resolution HAADF‐STEM image along the [110] axis (scale bar, 0.5 nm) and corresponding diffraction pattern (scale bar, 0.2 nm^−1^). g) The line profile along the Br─Cs column reveals the atomic arrangement for a Br─Br spacing of 0.54 and 0.42 Å, indicative of the orthorhombic perovskite framework. h) Simulated structure of CsPbBr_3_ along the [001] axis. i) Atomic‐resolution HAADF‐STEM image along the [001] zone axis (scale bar, 0.5 nm) and corresponding diffraction pattern (scale bar, 0.2 nm^−1^). j) The line profile along Pb─Br column indicates the octahedral tilting along the c‐axis.

**Figure** **1**a shows the low‐dose 4D‐STEM reconstructed virtual dark field (vDF) images that directly visualize the size and spatial distribution of CsPbBr_3_ PeQDs. The PeQDs exhibit a median size of 6.5 nm, with size distribution shown in Figure S1 (Supporting Information). Such uniformity is crucial for achieving homogeneous optical properties, especially in all‐inorganic PeQDs, where size variation significantly broadens emission spectra.^[^ ^25^ ^]^ High‐resolution STEM‐energy dispersive spectroscopy (EDS) mapping of a single QD (Figure 1b) confirms compositional uniformity and stoichiometric composition, without detectable phase segregation, validating the quality of the PeQDs. This is consistent with the high structural homogeneity observed in the scanning electron diffraction (SED) pattern (Figure S2, Supporting Information) and XRD pattern (Figure S3, Supporting Information), where the degradation byproducts PbBr_2_ and Pb clusters are not detected. Figure 1c shows the PL spectrum of pristine CsPbBr_3_ QDs, revealing a sharp emission peak at 514 nm consistent with quantum‐confined green emission with a high PLQE of 96.7% (Figure S4, Supporting Information). The narrow linewidth of 20 nm confirms structural homogeneity within the ensemble and minimal inhomogeneous broadening. Time‐resolved photoluminescence (TRPL) measurements using time‐correlated single photon counting (TCSPC) (Figure 1d) further indicate a long average PL lifetime of ≈9.1 ns, consistent with the PLQE measurement.

High‐resolution HAADF‐STEM imaging provides critical insights into the local atomic structure, distinguishing the orthorhombic phase from cubic and tetragonal phases. Figure 1e,f shows imaging along the [110] zone axis demonstrating a well‐ordered perovskite framework with distinct atomic‐scale features. The interatomic distances for Br–Br are measured to be 0.54 Å, 0.42 Å and 0.28 Å for Cs–Cs (Figure 1g). These subtle structural distortions from the cubic structure precisely match the orthorhombic symmetry. Additionally, the distance between the closest Cs–Cs atoms exhibits slight variations, specifically 0.59 and 0.62 nm, consistent with theoretical simulations. Along the [001] zone axis (Figure 1h,i), we directly image characteristic octahedral tilting of the orthorhombic CsPbBr_3_ structure, highlighting the structural plasticity. The PbBr_6_ octahedra exhibit a consistent spacing of ≈0.60 nm (Figure 1j). Understanding these subtle atomic‐scale features and the tilting of coordination polyhedra is crucial for establishing a structural reference for temperature‐induced phase transitions and defect dynamics. These distortions not only influence local strain fields but also affect charge transport and defect migration, directly impacting the optoelectronic performance and long‐term stability of perovskite LEDs.^[^ ^26^, ^27^ ^]^

By resolving sub‐ångström distortions at room temperature, previously inaccessible with conventional electron microscopy due to inherent beam sensitivity and weak scattering contrast, we provide fundamental insights into the structural adaptability of CsPbBr_3_ QDs, providing the foundations to further understand the potential phase evolution and defect healing mechanisms under external stimuli.

### 2.2 Temperature‐Driven Structural Evolution And Phase Transition

**Figure 2:**
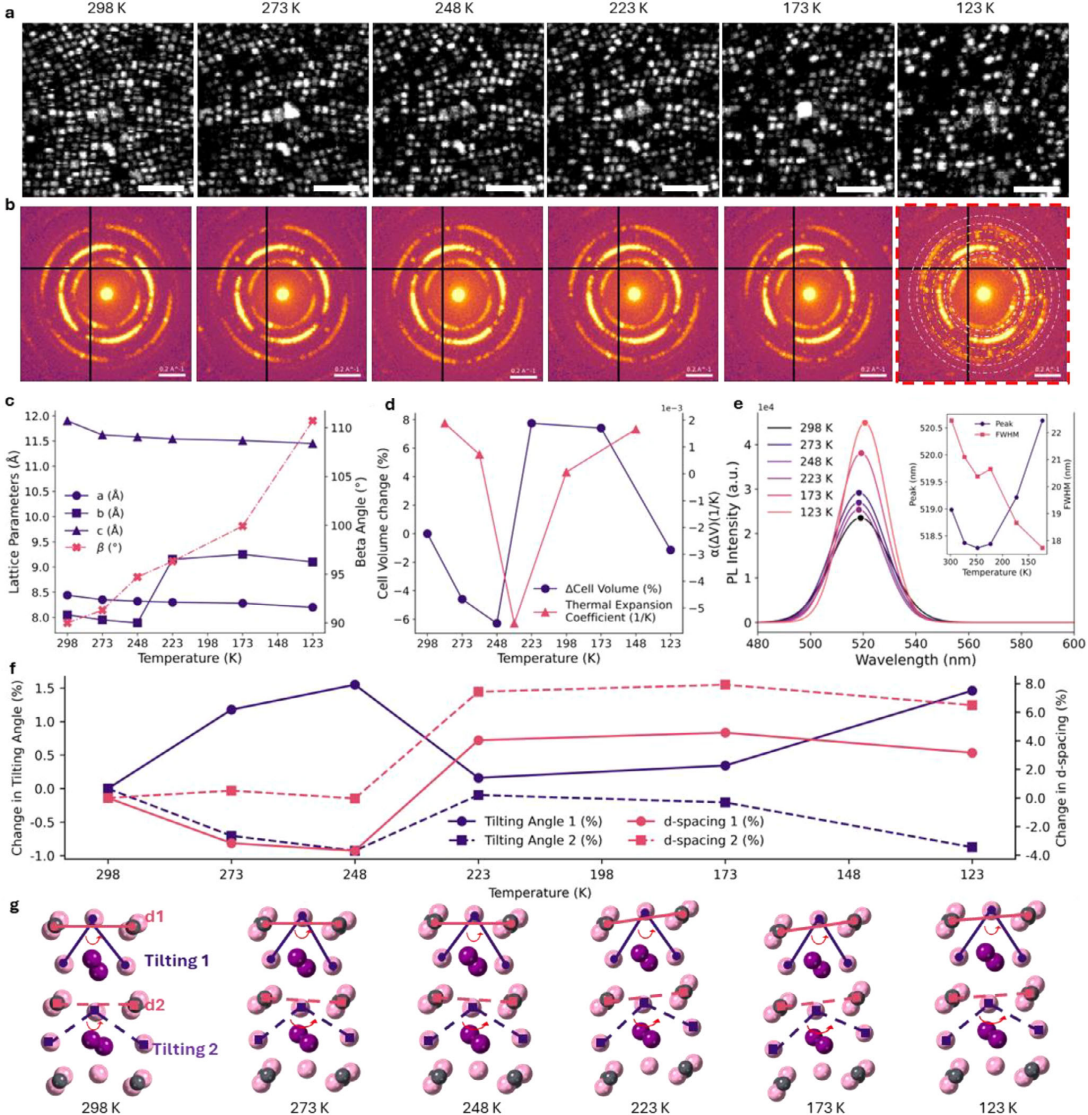
Temperature‐dependent structural and optical evolution of CsPbBr_3_ QDs during in situ cryogenic cooling. a) Virtual dark field (vDF) images acquired from the same sample area at different temperatures: 298, 273, 248, 223, 173, and 123 K. Scalebar: 50 nm. b) Representative diffraction patterns from each temperature point, showing emerging interspacing reflections at lower temperatures, consistent with symmetry lowering. c) Temperature dependence of refined lattice parameters and β angle, demonstrating the gradual structural transition from orthorhombic to monoclinic symmetry upon cryo‐state. d) Evolution of the unit cell volume with temperature, illustrating thermal expansion behavior and associated changes in thermal expansion coefficient. e) Temperature‐dependent PL spectra, highlighting changes in peak position and FWHM with temperature; the inset summarizes the peak shift and linewidth evolution. f) Evolution of octahedral tilting angles (Tilting 1 and Tilting 2) and inter‐octahedral spacings (d‐spacing 1 and d‐spacing 2) as a function of temperature, highlighting anisotropic strain accommodation, abrupt reorganization at the transition point, and structural stabilization in the low‐temperature monoclinic phase. g) Schematic illustration summarizing the temperature‐induced evolution from orthorhombic to monoclinic symmetry via progressive octahedral tilting and lattice distortion.

The structural evolution of PeQDs across the cryogenic temperature regime was investigated using 4D‐STEM reconstructed virtual dark‐field (vDF) imaging, electron diffraction patterns, and temperature‐dependent PL. Oleic acid‐capped QDs were deposited on an electron‐transparent substrate from solution, followed by pre‐baking at 50 °C to remove residual solvent. The specimen was loaded onto a cryo‐holder and transferred to the TEM vacuum chamber. Structural 4D‐STEM datasets were collected at cryogenic temperatures of 298, 273, 248, 223, 173, and 123 K, following a uniform cooling rate of 1 K min^−1^ and a 30‐min equilibration period at each step. At ambient temperature (298 K, 10^−4^ Pa), vDF images (**Figure** **2**a) exhibit uniform diffraction contrast, consistent with a highly ordered orthorhombic (Pnma) structure. The cryo‐state at 273 K introduces subtle contrast variations, indicating the onset of localized symmetry‐breaking lattice distortions. These effects intensify below 248 K, where pronounced contrast loss for [001]‐oriented QDs with the c‐axis aligned parallel to the electron beam. This reveals increased octahedral tilting and reduced lattice coherence. At 223 K, these distortions become widespread, signifying a lower symmetry transition (Figure S5, Supporting Information). Figure 2b gives electron diffraction patterns that provide further complementary evidence of this progression. While diffraction patterns at 298 and 273 K retain orthorhombic symmetry, the appearance of new satellite reflections below 248 K indicates the formation of superlattice periodicities due to correlated octahedral tilting and symmetry breaking.^[^ ^28^, ^29^ ^]^ The structural refinement of these diffraction patterns (Figure 2c; Table S1, Supporting Information) reveals a gradual phase transition from orthorhombic Pnma to monoclinic P2_1_/c symmetry, with the monoclinic β angle increasing from 90° to 110.7° at 123 K, serving as a quantitative marker of symmetry distortion in the cryogenic regime. The temperature‐dependent evolution of unit cell volume (Figure 2d) highlights three distinct regimes. Above 248 K, the structure exhibits linear thermal contraction. Between 248 and 223 K, the volume stabilizes, consistent with a lattice rearrangement in which octahedral tilts become cooperatively locked into a lower‐energy configuration. This intermediate regime precedes the full monoclinic transformation, which is completed below 223 K. These structural stages correlate with the diffraction contrast changes in Figure 2a–c. Differential scanning calorimetry (DSC) at ambient pressure (Figure S6, Supporting Information) pinpoints the phase transition at 227 K. A higher latent heat observed in cycled DSC measurements suggests reduced disorder and strain during the first conditioning cryo‐cycle.

The correlation between the observed structural evolution and optical properties of the QDs is further elucidated by temperature‐dependent PL spectroscopy at vacuum conditions (Figure 2e). Initial cryogenic cooling from 298 to 273 K induces a progressive blue shift in PL emission, consistent with bandgap widening arising from thermal contraction and reduced electron‐phonon interactions.^[^ ^30^ ^]^ This is accompanied by enhanced PL intensity, suggesting the suppression of non‐radiative recombination (Figure S7, Supporting Information). Between 248 and 223 K, the PL peak redshifts slightly with the FWHM modestly broadens. These variations appear to be associated with the orthorhombic to monoclinic phase transformation occurring within this temperature range. Specifically, thermal contraction and increased octahedral tilting are accompanied by the emergence of localized strain domains and structural disorder that contribute to spectral broadening and exciton quenching.

Below 223 K, a drastic decrease in PL intensity and rapid bandgap widening are observed, reflecting the emergence of monoclinic ferroelastic domains.^[^ ^31^, ^32^ ^]^ These domains significantly disrupt electronic coherence by introducing local strain fields and altering Pb–Br–Pb bond angles, thereby reducing orbital overlap and carrier mobility, which in turn decreases radiative recombination efficiency.^[^ ^32^ ^]^ Crucially, the absence of significant PL linewidth broadening across the entire temperature range suggests a diffusion‐less (Martensitic‐like) transformation, where the phase transition occurs through cooperative atomic rearrangements rather than random diffusion‐driven processes.^[^ ^33^, ^34^, ^35^ ^]^ This indicates that the perovskite lattice dynamically accommodates thermal stress without introducing significant disorder, preserving optical coherence (Figure 2e, inset). Analysis of octahedral tilting angles and interplanar d‐spacings along the [001] zone axis at in‐plane and out‐of‐plane directions (Figure 2f) provides direct insights into the structural degradation mechanisms. Between 298 and 248 K, anisotropic octahedral tilting is observed, with in‐plane tilting increasing moderately (1.55%) and out‐of‐plane tilting slightly decreasing (0.93%). Below 248 K, both tilting angles and d‐spacings show abrupt changes, confirming the onset of the symmetry‐lowering phase transition. From 248 to 223 K, both d‐spacings increase sharply (by 4.04% and 7.43%), consistent with a sudden reorganization of the framework as the monoclinic phase develops. Below 223 K, the angles and d‐spacings gradually stabilize, marking the completion of the transition. A systematic illustration of temperature‐dependent evolution in tilting angles and inter‐octahedral spacings is shown in Figure 2g. Collectively, these findings establish the first direct atomic‐scale visualization of the cryogenic phase transition in CsPbBr_3_ QDs, elucidating how symmetry breaking and lattice distortions govern their structural dynamics and optical properties.

### 2.3 Cryo‐Cycling Induced Strain Relaxation and Orientation Reordering

**Figure 3:**
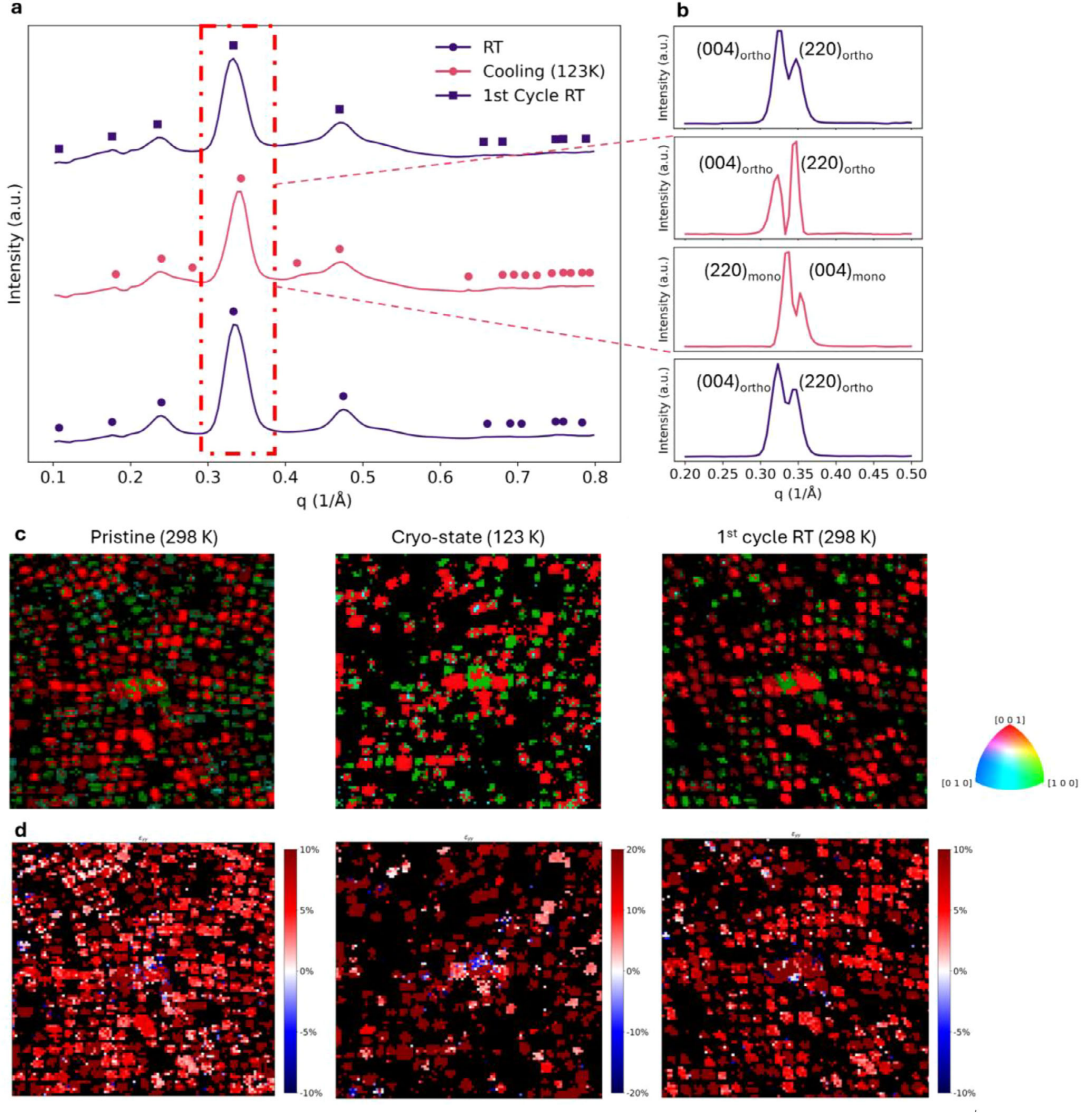
Structural relaxation, strain release, and orientation recovery after 1st cryo‐cycle. a) Summed 1D electron diffraction patterns of the sample at 298 K (pristine), 123 K (cryo‐state), and 1st cycle RT to 298 K at 1 K min^−1^. b) The (004) and (220) diffraction peaks extracted from 1D radial profiles(highlighted in red), analyzed via the NMF algorithm to resolve peak splitting associated with orthorhombic and monoclinic phases. c) Out‐of‐plane orientation maps, showing increasing orientation heterogeneity during cooling and a return to more uniform [001] alignment after 1st cryo‐cycle. d) Lateral strain maps at the same region in c).

To investigate the strain within CsPbBr_3_ QDs during cryo‐cycling structural evolution, we analyzed spatial orientation and strain maps derived from Euler angle information in the 4D‐STEM dataset. The Automated Crystal Orientation Mapping (ACOM) algorithm was used to quantify grain orientation and misalignment with high spatial resolution. **Figure** **3**a shows summed 1D radial diffraction patterns. At 123 K, diffraction peaks shift to higher q values due to thermal contraction. The emergence of satellite peaks between primary reflections indicates the presence of a monoclinic phase, with a lower symmetry featuring c‐axis tilting induced periodic peaks. Quantitative analysis of diffraction intensities reveals an increase in satellite peak intensity upon cryogenic cooling. The satellite peak intensity is partially reduced upon reheating, suggesting partial lattice recovery. Upon reheating to 298 K, the peaks shift back to lower q values, reflecting partial strain relaxation and lattice expansion. Close inspection of the (004) and (220) reflections (Figure 3b) confirms this trend, with the initial peak splitting into two components for each plane, separating (004) and (220) for monoclinic phase and orthorhombic phase, respectively at 123 K, indicating monoclinic distortion and octahedral tilting, followed by partial recombination into two narrower components after reheating, suggesting incomplete crystallographic recovery.

Orientation mapping (Figure 3c) reveals that most QDs exhibit a preferred out‐of‐plane [001] zone axis in the pristine state, consistent with partial epitaxial registry to the substrate (Figure S11, Supporting Information). Misorientation maps (Figures S12,S13, Supporting Information) show that 47.5% of grains initially align within a few degrees of [001], favoring efficient charge transport. Upon cryogenic cooling, the orientation distribution broadens significantly in both in‐plane and out‐of‐plane zone axes, with many grains tilting away from [001], consistent with diffraction contrast loss in vDF image at 123 K (Figure 2a). This is attributed to cryo‐induced internal stress and surface destabilization. Upon reheating, a greater proportion of the QDs align with the [001] parallel to the electron beam, which confirms a propensity for the orthorhombic phase to align along the low‐energy c‐axis. This is accompanied by partial defect healing and lattice relaxation. However, some QDs remain misoriented, likely due to kinetic limitations associated with residual strain at grain boundaries, which hinders complete orientation recovery.

Figure 3d shows the changes in local strain fields, consistent with the evolution in orientation. In the pristine state, the out‐of‐plane ε_yy_ strain is relatively homogeneous, with mild compressive and tensile strain (±5%) near grain boundaries. At 123 K, strain heterogeneity increases sharply, with tensile ε_yy_ strain exceeding 20% in localized regions, particularly at grain boundaries and the sample surface. Cryo‐induced strain arises from differential thermal contraction between grains, monoclinic phase formation, and ligand detachment, which disrupts atomic coordination and increases surface energy. The evolution of the ε_xx_ strain follows a similar trend (Figure S10, Supporting Information). Initially, the ε_xx_ strain maps show regions of up to 10% tensile strain, with some grains exhibiting mild compressive strain (<5%). At 123 K, tensile strain increases, with more grains reaching 20% and almost no compressive strain regions. This strain buildup exacerbates orientation disorder by driving rotational misalignment. After reheating, the ε_xx_ and ε_yy_ strains partially relax, with some regions returning to nearly 0% strain and large regions holding a more homogeneous strain distribution, indicating partial internal stress release between grain boundaries.

The observed strain and orientation disorder evolution is influenced by surface ligand behavior. In the pristine state, oleic acid ligands passivate QD surfaces, suppressing halide vacancies and minimizing surface strain. DSC confirms that oleic acid undergoes a phase transition near 286K, within the studied cryo‐range (Figure S6, Supporting Information). After 1st cryo‐cycle at RT, ligand detachment exposes under‐coordinated Pb and halide sites, promoting surface vacancies and increasing ε_xx_ strain. Upon reheating, increased ionic mobility facilitates partial surface reconstruction, as indicated by the changes in diffraction patterns (Figure S8, Supporting Information). This includes partial recombination of split peaks with higher diffraction intensity and narrower amorphous rings. However, not all defects are healed, and some remain, particularly those at grain boundaries, leading to persistent misorientation and localized strain pockets.

### 2.4 Cryogenic Treatment Induced Deep Defect Healing

**Figure 4:**
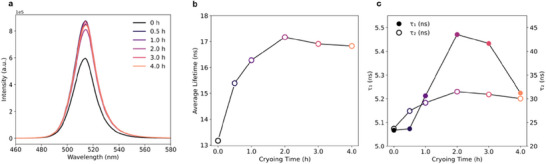
Evolution of photoluminescence and carrier dynamics in CsPbBr_3_ under varying cryogenic durations. a) PL spectra of CsPbBr_3_ QDs in hexane with a concentration of 0.5 mg mL^−1^ after different durations of cryo‐treatment at 77 K. An initial increase in PL intensity is observed, attributed to defect passivation, followed by a decrease in intensity upon prolonged freezing. b) Time‐resolved photoluminescence (TRPL) measurements reveal a corresponding increase in carrier lifetime during moderate freezing, followed by a lifetime reduction after extended cryogenic exposure. c) Evolution of fast (τ_1_) and slow (τ_2_) photoluminescence decay components as a function of cryogenic time.

Inspired by the abovementioned study, we further utilize the cryogenic treatment as an effective post‐synthesis method to enhance the optical performance. We propose and prove that this method can achieve defect healing and strain relaxation by a proper treatment duration. We employed PL spectra and TCSPC lifetime CsPbBr_3_ QDs to study how different cryogenic post‐synthesis durations in liquid nitrogen (LN_2_) at 77 K with the same reheating duration to RT will influence the optical performance of CsPbBr_3_ QDs. **Figure** **4** demonstrates that our controlled cryo‐treatments could significantly influence optical properties, defect behavior, and structural relaxation in these PeQDs. By changing the cryogenic durations from 0.5 to 12 h, the PL emission peak of CsPbBr_3_ QDs remains centered at around 514 nm, confirming consistent bandgap and chemical composition without detectable phase segregation or decomposition during the cryo‐treatments (Figure 4a; Figure S14 and Table S2, Supporting Information). The FWHM also remains constant (18 nm), confirming internal crystal structure integrity without substantial structural disorder or size distribution broadening. PL intensity initially increases with cryo‐treatment time, peaking at 0.5–1 h, then gradually decreases with longer exposures. The initial enhancement is attributed to the suppressed thermal vibrations at cryogenic temperatures, enabling atomic rearrangements that heal defects such as halide vacancies and undercoordinated ions. These changes reduce non‐radiative recombination and localized strain. However, cryo‐treatment beyond 2 h leads to a decrease in PL intensity, particularly after overnight exposure, suggesting new defect formation induced by thermal contraction, leading to lattice distortion, mechanical stress‐induced surface cracking during the prolonged low‐temperature treatment.

PL lifetime measurements by TCSPC further prove this non‐monotonic behavior varying cryogenic treatment durations (Figure S15, Supporting Information), offering direct insight into the dominant recombination dynamics (Figure 4b,c). The PL lifetime of CsPbBr_3_ QDs increases from 13.2 ns in the pristine state to a maximum of 17.2 ns at 2 h of cryo‐stage before decreasing to 12.6 ns after extended cryo‐treatment (Figure S16, Supporting Information). This trend, measured at RT after 8 h of relaxation, aligns well with surface recombination center healing and strain relaxation, after the orthorhombic‐monoclinic transition, consistent with enhanced PL intensity at intermediate cryo‐treatment durations.

To further disentangle the recombination pathways, bi‐exponential fitting was applied (Table S2, Supporting Information), yielding fast (τ_1_) and slow (τ_2_) lifetime components, both of which exhibit non‐monotonic trends (Figure 4c). The short lifetime τ_1_, attributed to carrier recombination via deep‐trap states such as halide or lead vacancies,^[^ ^36^ ^]^ increases from 5.1 to 5.5 ns at 2 h before declining to 4.7 ns after prolonged treatment. Simultaneously, the longer τ_2_ component, associated with excitonic radiative recombination, increases from 23.7 to 31.5 ns at 2 h and then decreases to 22.3 ns. This behavior suggests that moderate cryogenic treatment (2 h) and cycle to RT recovery, partially heal these deep traps likely by promoting strain relaxation. This post‐synthesis treatment will also enhance the radiative lifetimes. However, extended cryogenic exposure appears to reintroduce defects or micro‐strain and shorten both τ_1_ and τ_2_. Notably, the amplitude of the fast component also decreases at 2 h, further supporting the reduced contribution of trap‐assisted recombination at this point. Overall, these findings demonstrate that a 2 h cryogenic post‐synthesis treatment most effectively enhances the optical performance of CsPbBr_3_ quantum dots.

Importantly, these optical improvements are only fully realized after a relatively long recovery (>8 h) at RT to allow the deep trap healing (Figure S16, Supporting Information). This long recovery period enables lattice relaxation and atomic reorganization, completing defect healing processes initiated at 77 K. Without sufficient thermal activation, defect states remain kinetically trapped, and exciton lifetimes show limited enhancement. Thus, RT recovery is an essential step to stabilize deep‐trap healed configurations and maximize the optical performance of QDs. Coupled with structural insights from the STEM investigation, our findings highlight cryogenic processing as a promising strategy to enhance structural uniformity, deep defect healing, and optical performance in PeQDs for achieving stable, high‐efficiency perovskite optoelectronics.

## 3 Conclusion

In summary, we present direct nanoscale evidence of an unreported low‐temperature orthorhombic‐to‐monoclinic phase transition in CsPbBr_3_ QDs, distinct from the bulk behavior. Using sub‐ångström HAADF‐STEM, we resolve intrinsic octahedral tilting at room temperature, showing that QDs begin in a pre‐tilted, low‐symmetry state that predisposes them to monoclinic distortion upon cryogenic cooling. Complementary temperature‐dependent 4D‐STEM diffraction mapping and PL spectroscopy reveal that cryogenic cooling induces severe strain heterogeneity and surface defect formation, amplified by ligand detachment. Strain concentrations exceeding 20% emerge at grain boundaries and surfaces. However, upon 1st cryo‐cycle, surface strain partially relaxes, and some defects heal, while deeper grain‐boundary defects remain kinetically trapped. We also provide a cryogenic post‐synthesis treatment with an optimal cryogenic window of 1–2 h and recovery at RT. Our novel method is proven to heal the deep traps and enhance the radiative recombination. Prolonged exposure will cause irreversible degradation and a decrease in the optical performance of CsPbBr_3_ QDs. These findings establish a direct link between thermal cycling, defect dynamics, and optical performance in PeQDs, offering a scalable, low‐cost strategy to improve structural and optical stability. More broadly, the insights into phase flexibility and strain accommodation offer guiding principles for defect engineering in other strain‐sensitive semiconductor systems.

## 4 Experimental Section

### Synthesis of CsPbBr_3_ QDs

The CsPbBr_3_ QDs have been synthesized using a method from the recent work with modifications.^[^ ^37^ ^]^ Cs_2_CO_3_ (0.133 g), OA (0.42 mL), and ODE (5 mL) were added into a 50 mL three neck round‐bottom flask and heated at 140 °C under N_2_ flow until fully dissolved to form Cs‐oleate precursor. Separately, PbBr_2_ (0.1835 g), ODE (15 mL), OA (1.5 mL), and OAm (1.5 mL) were added into a 50 mL three neck round‐bottom flask. The reactants were stirred and heated to 120 °C under N_2_ flow for 1 h until fully dissolved. The flask is then heated up to 180 °C. 1 mL of Cs‐oleate is then swiftly injected into the reaction mixture. After 15 s, the flask is placed in an ice‐water bath. After cooling down to room temperature, the resulting CsPbBr_3_ QDs are precipitated out by centrifuging at 11 617 × *g* for 10 min. The precipitate is then dispersed in 7 mL of hexane. The solution is further purified by centrifugation and filtration to get rid of large QDs, and stored in a glass vial for future usage.

### Characterization

UV‐vis absorption spectra were obtained using a Shimadzu UV2450/UV3600 spectrometer in solution at room temperature. PL spectra are obtained by Andor Kymera 328i spectrometer housing a DU420A Silicon CCD detector. PLQE measurement was carried out using a Spectralon‐coated integrating sphere. Measurements were taken at room temperature using CW laser diodes as the excitation source. Light from the experiment was collected using an optical fiber connected to an Andor Kymera 328i spectrometer housing a DU420A Silicon CCD detector. Setup calibration was performed using a Bentham 610 QTH calibration source. XRD measurement was performed on a Bruker D8 Discover with Cu Kα X‐ray source.

### Time‐Correlated Single Photon Counting (TCSPC) Measurements

The CsPbBr_3_ NC solution was photoexcited using a 407 nm pulsed laser with a pulse width <500 ns, at a repetition rate of 1 kHz. Photons emitted from the sample were collected by a silicon‐based single‐photon avalanche photodiode. The instrument response function has a lifetime of ≈0.2 ns.

### Sample Preparation for Optical Characterization

For all optical measurements, CsPbBr_3_ QDs were dispersed in hexane at a concentration of 0.5 mg mL^−1^. The dispersion was aliquoted into individual centrifuge tubes, each subjected to a cryogenic treatment by immersion in liquid nitrogen (77 K) for durations ranging from 30 min to 12 h. Following cryo‐exposure, all samples were allowed to recover under ambient conditions for 8 h before measurement. This recovery step was necessary, as samples measured immediately after cooling exhibited lower PL intensity and shorter lifetimes than those allowed to thermally relax. The relaxation time ensured the stabilization of defect states and accurate comparison across cryo‐treatment durations.

### 4D‐STEM Data Collection

During SED microscopy, a 2D electron diffraction pattern was measured at every probe position of an electron beam in STEM mode. SED data were acquired on the JEOL ARM300CF E02 instrument at ePSIC (Diamond Light Source, Didcot‐Oxford, UK). A monolithic Merlin/Medipix direct electron detector was used to acquire fast, low‐dose SED. These direct electron detectors allow for better SNR under lower doses due to the superior quantum efficiency compared to traditional CCD. The detector was set to 6‐bit, to maintain the targeted electron fluence and fast acquisition readout rate. An acceleration voltage of 200 keV, nanobeam alignment (convergence semi‐angle ≈1 mrad), probe current ≈1.3 pA, scan dwell time 0.6 ms, and camera length 40 cm were used. An estimated electron fluence of <7 e^−^Å^−2^ per frame was acquired when approximating the beam cross‐section as a disk. This accumulated dose is lower than the previously reported damage threshold for hybrid perovskite compositions.^[^ ^38^, ^39^ ^]^ Post‐processing of SED diffraction data was done using py4DSTEM 0.14.14 (an open‐source set of Python tools for processing and analysis of 4D scanning transmission electron microscopy (4D‐STEM) data).^[^ ^40^ ^]^

In situ cryo‐cooling was conducted with a Fischione single‐tilt LN_2_ holder. The sample was cooled from room temperature to 123 K at a controlled rate of 1 K min^−1^. The beam was blanked during cooling. The temperature stabilization time was 30 min at each step before acquisition.

Lattice parameters were refined by fitting the observed d‐spacings extracted from 1D radial intensity profiles to theoretical spacings derived from crystallographic equations, accounting for symmetry constraints specific to orthorhombic (Pnma) and monoclinic (P2_1_/c) structures. Candidate planes were assigned based on the strongest observed peaks, and extinction rules were used to validate space group assignments. The refinement was carried out using nonlinear least‐squares optimization to minimize the residuals between measured and calculated d‐values. To quantify the goodness of fit, both the Mean Squared Error (MSE) and Root Mean Squared Error (RMSE) were calculated. Lower RMSE values indicated stronger agreement between the model and the experimental dataset. Systematic residual patterns and mismatch of forbidden reflections were used to distinguish between orthorhombic and monoclinic assignments, with the emergence of new low‐temperature peaks and β‐angle distortion supporting a monoclinic phase at 123 K.

To resolve subtle symmetry changes in diffraction profiles, principal component analysis (PCA) and non‐negative matrix factorization (NMF) were applied to the 1D radial electron diffraction intensity profiles obtained at room temperature (RT), cryogenic state (123 K), and post‐first‐cycle RT. These techniques enabled data‐driven decomposition of diffraction intensity variations and identification of key features linked to structural transitions. Notably, peak splitting of the (004) and (220) reflections was resolved in the cryogenic‐state dataset, distinguishing monoclinic distortion from the single‐peak signatures observed in the pristine orthorhombic phase. Orientation and strain mapping were carried out using py4DSTEM‐based scripts. A simulated zone axis library derived from orthorhombic CsPbBr_3_ was used to index local diffraction patterns to extract Euler angles, from which in‐plane and out‐of‐plane orientation maps were constructed. To evaluate crystal orientation changes, misorientation concerning the global [001] axis was computed using a custom Python script that calculates angular deviations based on the extracted Euler angles. Strain tensor components (ε_xx_, ε_yy_, ε_xy_) were determined from Bragg peak shifts using sub‐pixel Gaussian fitting. This methodology enabled high‐resolution spatial mapping of strain localisation and orientation reordering across cryogenic transitions.

Structural models of orthorhombic and monoclinic CsPbBr_3_ were simulated using CrystalMaker software. The program was used to visualize atomic arrangements, compare simulated and experimental diffraction features, and evaluate octahedral tilting distortions associated with the phase transition.

### STEM‐HAADF Image and STEM‐EDX Spectrum

High‐resolution HAADF‐STEM was performed on a TF Spectra 300 (200 kV, 17.1 mrad convergence angle, 115 mm camera length, 1 µs dwell time, 1 pA current). Collection angles: 74.3 ± 0.3 to 233.3 ± 1.5 mrad. Images were acquired at 512 × 512 pixels over 10 frames with a 1 µs pixel dwell time. Drift‐corrected frame integration (DCFI) was applied to reconstruct a periodic image, enhancing spatial resolution. Post‐acquisition, a radial and high‐pass filter was applied to the reconstructed image to enhance sub‐angstrom feature visibility and improve the contrast of light‐element columns.

Energy Dispersive X‐ray Spectroscopy (STEM‐EDX) maps were acquired using four Super‐X detectors with a dwell time of 300 µs per pixel across 50 frames, achieving a spectral resolution of 10 eV per channel. STEM‐EDX spectrum images were collected using a beam current of 30 pA, a convergence angle of 24 mrad, and a camera length of 91 mm.

### DSC Measurement

DSC was performed with a TA Instruments DSC 2500 (2 K min^−1^) at ambient N_2_ atmosphere. CsPbBr_3_ QDs (2.628 mg) in hermetically sealed Al pans were cycled from 303.15 to 183.15 K. Enthalpy (ΔH) changes were extracted from baseline‐subtracted dQ/dT curves.

## Conflict of Interest

The authors declare no conflict of interest.

## Author Contributions

X.L. conceptualized the ideas under the supervision of C.D.; X.L. and Z.Y. designed the methodology of the cryo‐experiment; Z.Y. synthesized the perovskite quantum dots; X.L. and T.W. performed the in situ cryo‐experiment in ePSIC, DLS; Z.J. performed PL and TRPL measurements; S.C. performed temperature‐dependent PL and PLQE measurements; Y.T. performed DSC measurement and interpreted the data under the supervision of X.M.; B.X. performed TA measurement and interpreted the dataset under the supervision of A.R.; Y.L. performed XRD measurement; X.L. analyzed and interpreted all cryo‐datasets under the supervision of C.D.; X.L. interpreted the PL and TRPL data under the supervision of Z.Y.; X.L., C.D., and Z.Y. wrote the manuscript with input from all authors.

## Supporting information



Supporting Information

## Data Availability

The data that support the findings of this study are available from the corresponding author upon reasonable request.
