# Supplementary material for: Visualizing Strain‐Coupled Cryogenic Phase Transitions and Defect Dynamics in Perovskite Quantum Dots Using In Situ STEM
Source: Adv Sci (Weinh). 2025 Dec 7;13(11):e16496. doi: 10.1002/advs.202516496 (PMC12931179; doi:10.1002/advs.202516496)
Supplement: Supplementary file 1 — Supporting Information [file ADVS-13-e16496-s001.docx]

Supporting Information

Visualising Strain-Coupled Cryogenic Phase Transition and Defect Dynamics in Perovskite Quantum Dots Using In Situ STEM

Xinjuan Li^1^, Zhao Jiang^2^, Si Chen^2^, Yi Tang^1^, Bofeng Xue^2^, Tianhao Wu^1^, Yang Lu^2^, Xavier Moya^1^, Akshay Rao^2^, Zhongzheng Yu^2^*, Caterina Ducati^1^*

**Affiliations:**

^1^ Department of Materials Science and Metallurgy, University of Cambridge, Cambridge CB3 0FS, UK

^2^ Cavendish Laboratory, University of Cambridge, JJ Thomson Avenue, Cambridge CB3 0HE, UK

^*^E-mail address: zy338@cam.ac.uk (Z. Yu), cd251@cam.ac.uk (C. Ducati)

This file contains:

Figure. S1–16

Table S1–2


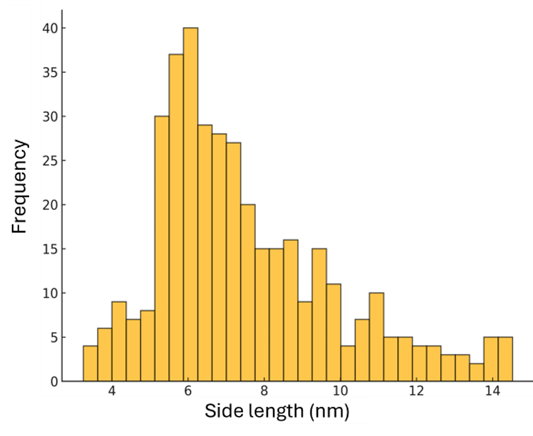


**Figure. S1. Size distribution of QDs at pristine conditions.** Histogram showing the side length distribution of CsPbBr_3_ QDs with native ligands, dispersed on a TEM grid without any post-treatment. The median size is 6.5 nm. Size statistics were obtained from image analysis of 406 individual quantum dots.


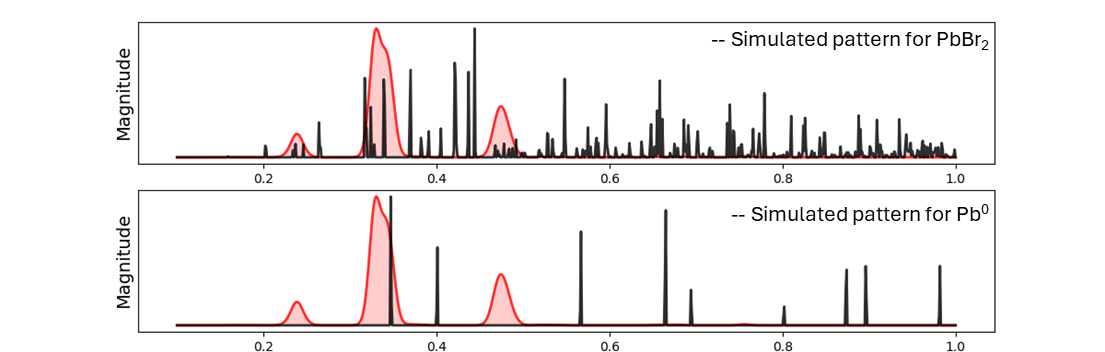


**Figure. S2.** The summed 1D radial profile for QDs at pristine condition, compared with the simulated diffraction pattern for PbBr_2_ and Pb^0^, considered as common degradation phases in CsPbBr_3_ composition. This confirms the initial homogeneity in QDs.


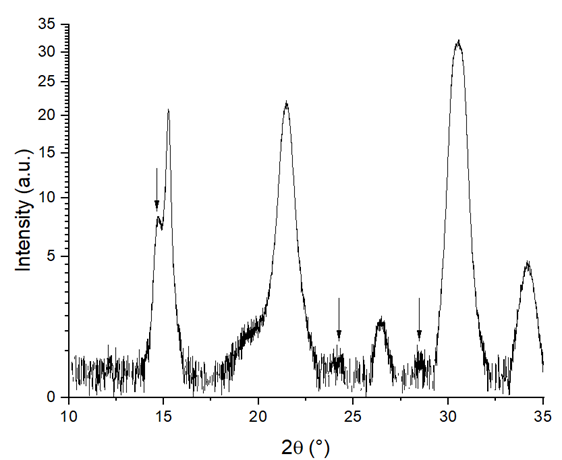


**Figure. S3. X-ray diffraction pattern of pristine CsPbBr_3_ QDs.** The diffraction peaks correspond to the orthorhombic crystal structure, with characteristic reflections(arrow marked) that clearly distinguish it from the cubic phase. The data confirm that the QDs retain their original orthorhombic phase before any external treatment.


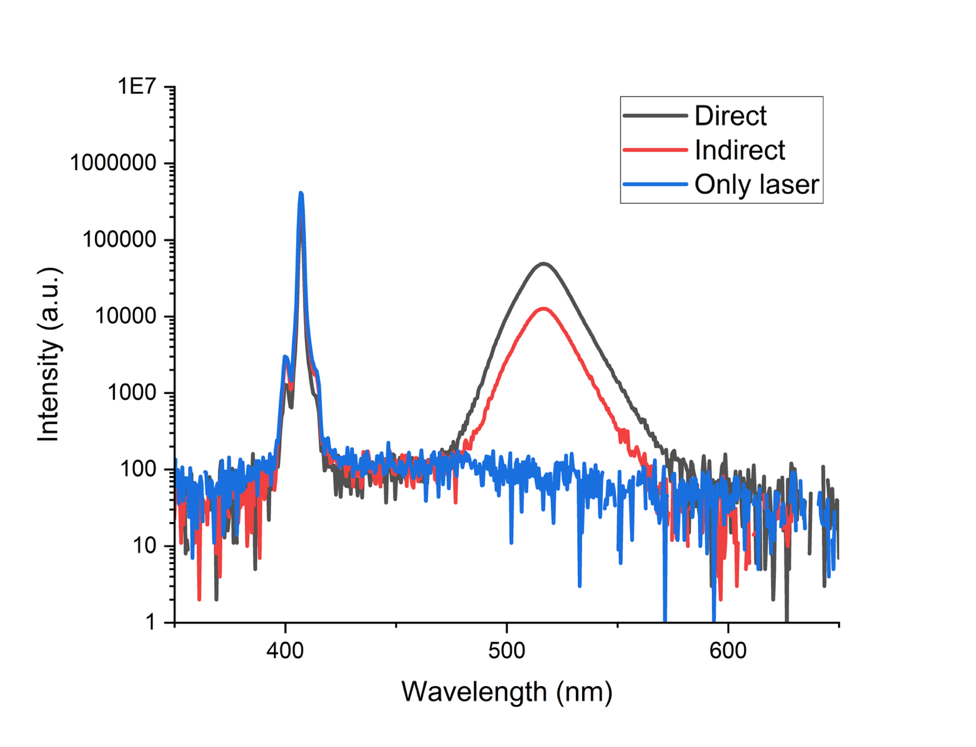


**Figure. S4.** The PLQY of CsPbBr_3_ quantum dots in solution with 96.7% on a logarithmic intensity scale.


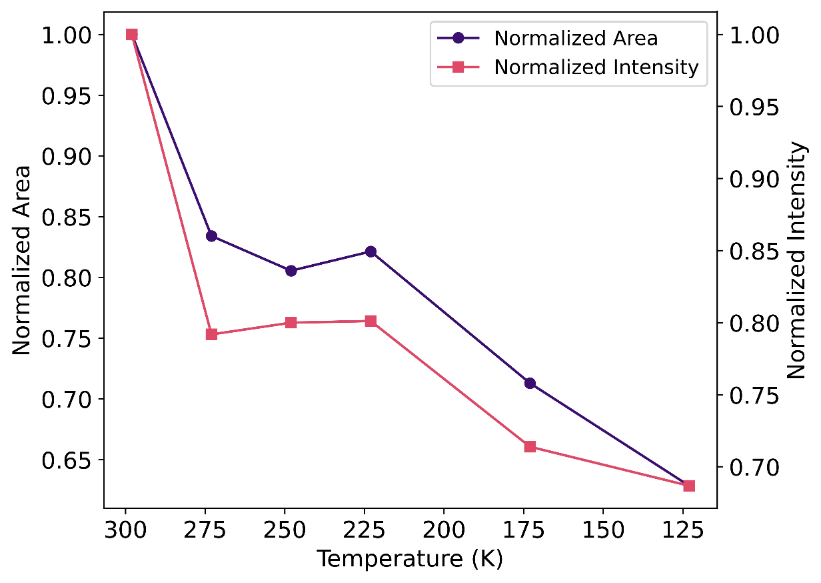


**Figure. S5.** The temperature-dependent changes in area and intensity extracted from reconstructed virtual dark-field (vDF) images acquired from the same region of CsPbBr_3_ QDs in Figure. 2a. Both the real-space area (blue) and mean intensity (red) are normalised to their values at room temperature (298 K). As temperature decreases, a progressive reduction in apparent area and image contrast is observed. This behaviour may arise from lattice contraction and reduced electron scattering cross-section upon cryogenic exposure.

**Table S1**. Lattice Parameters of CsPbBr₃ Quantum Dots Under Cryogenic Conditions


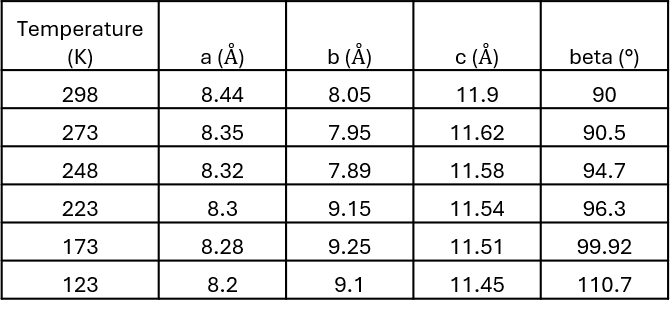


**Table S2**. Decay lifetime study of CsPbBr₃ QDs Under Cryogenic Conditions


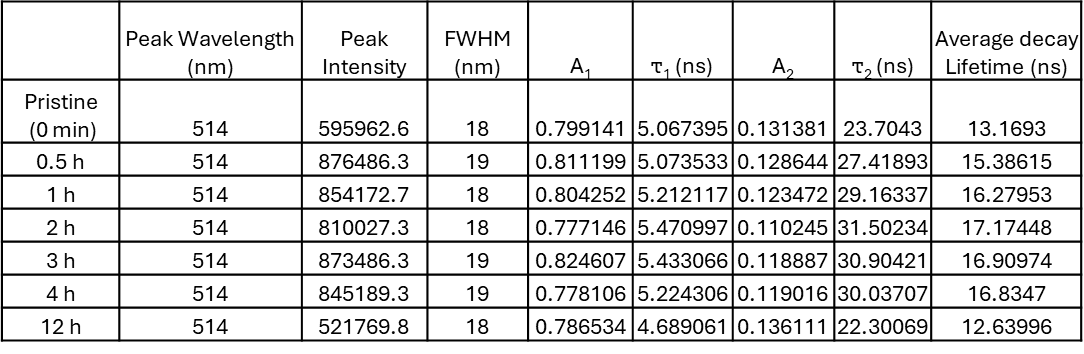


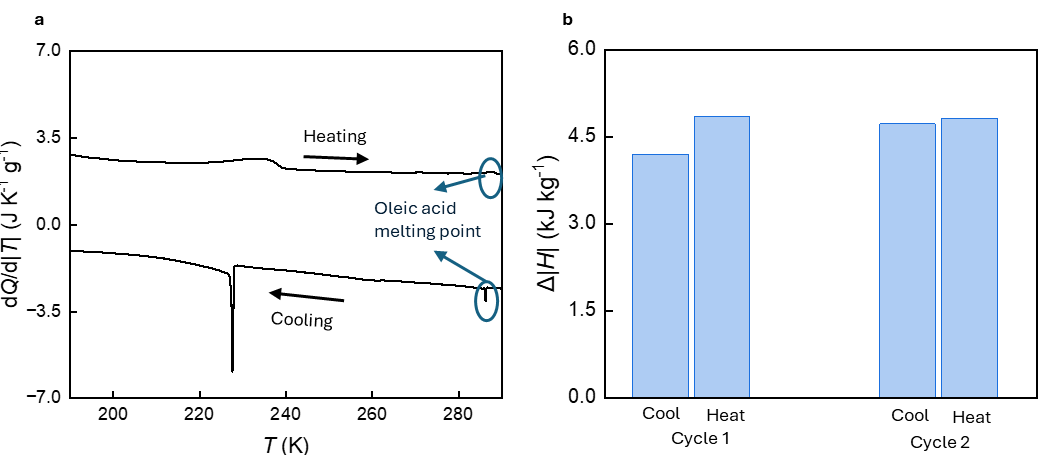


**Figure. S6. Differential scanning calorimetry of CsPbBr_3_ QDs in solution. a.** Cycled DSC traces of CsPbBr_3_ QDs capped with oleic acid reveal phase transition behaviour associated with both the QD core and surface ligands during the first two cryo-cycles. The features near 286K correspond to the melting point of oleic acid, indicating temperature-induced reorganisation of the ligand shell. The phase transition temperature of QDs is around 227K. b. The enthalpy change reflects the energy associated with these transitions and is normalised to the total mass of the QD and ligand mixture in solution. Samples were hermetically sealed in aluminium pans with lids to prevent solvent evaporation and maintain thermal integrity during measurement.


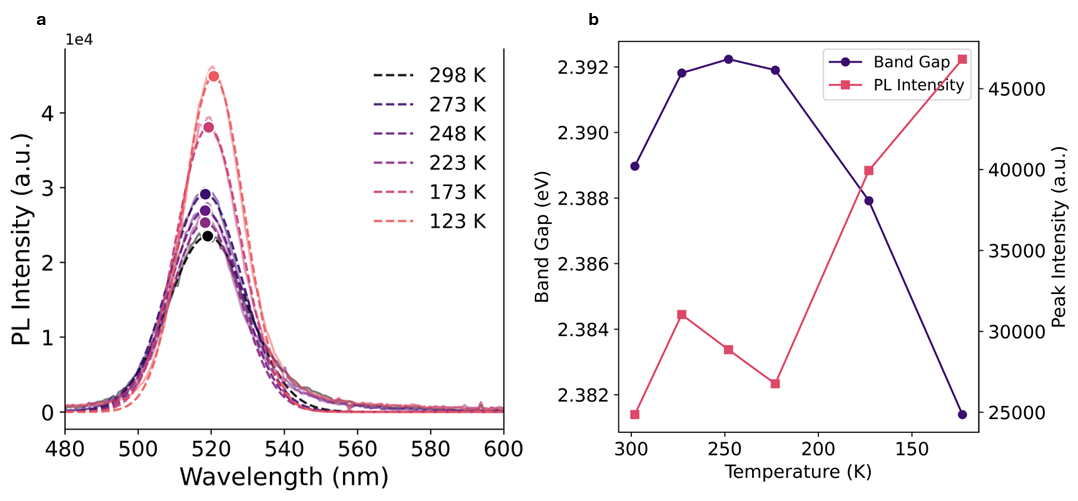


**Figure. S7. a.** The Gaussian fitting of temperature-dependent PL spectra. b. The extracted information of intensity changes and calculated band gaps with cooling temperatures.


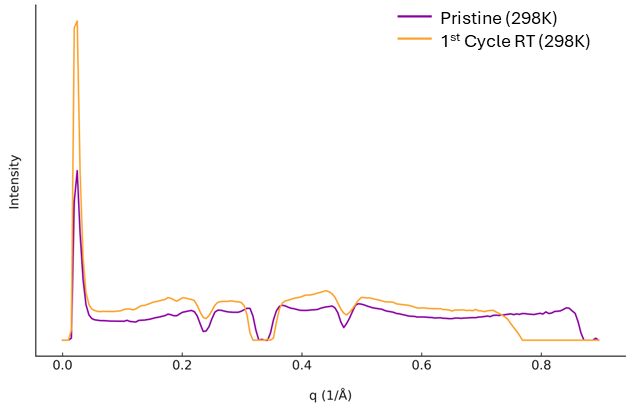


**Figure. S8. Structural reorganization of surface ligands upon thermal cycling.** 1D electron diffraction profiles related to regions surrounding QDs on a holey ultrathin carbon grid, minimising background contributions from the support film. Spectra at room temperature (purple) and after cooling to 123 K followed by reheating (orange) reveal distinct modulations and intensity changes, consistent with thermally induced reordering or densification of the ligand shell surrounding the QDs.


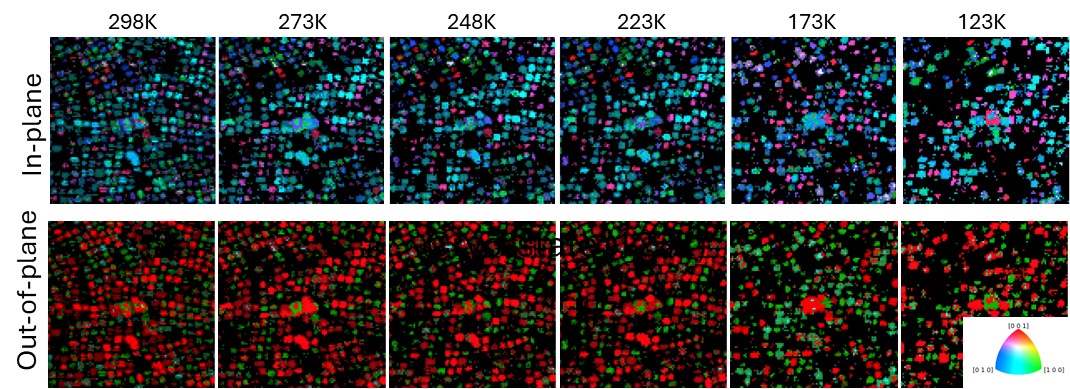


**Figure. S9.** (a) The in plane and out of plane orientation map generated from Euler angles in the same region in Figure 2(a).


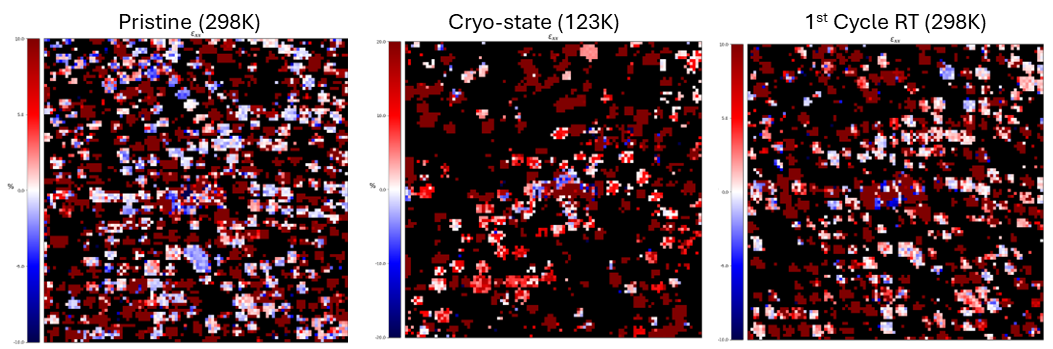


**Figure. S10.** (a) The in-plane strain map generated from Euler angles in the same region in Figure 3(d) for samples at pristine, cryo-state and 1^st^ cycle RT conditions.


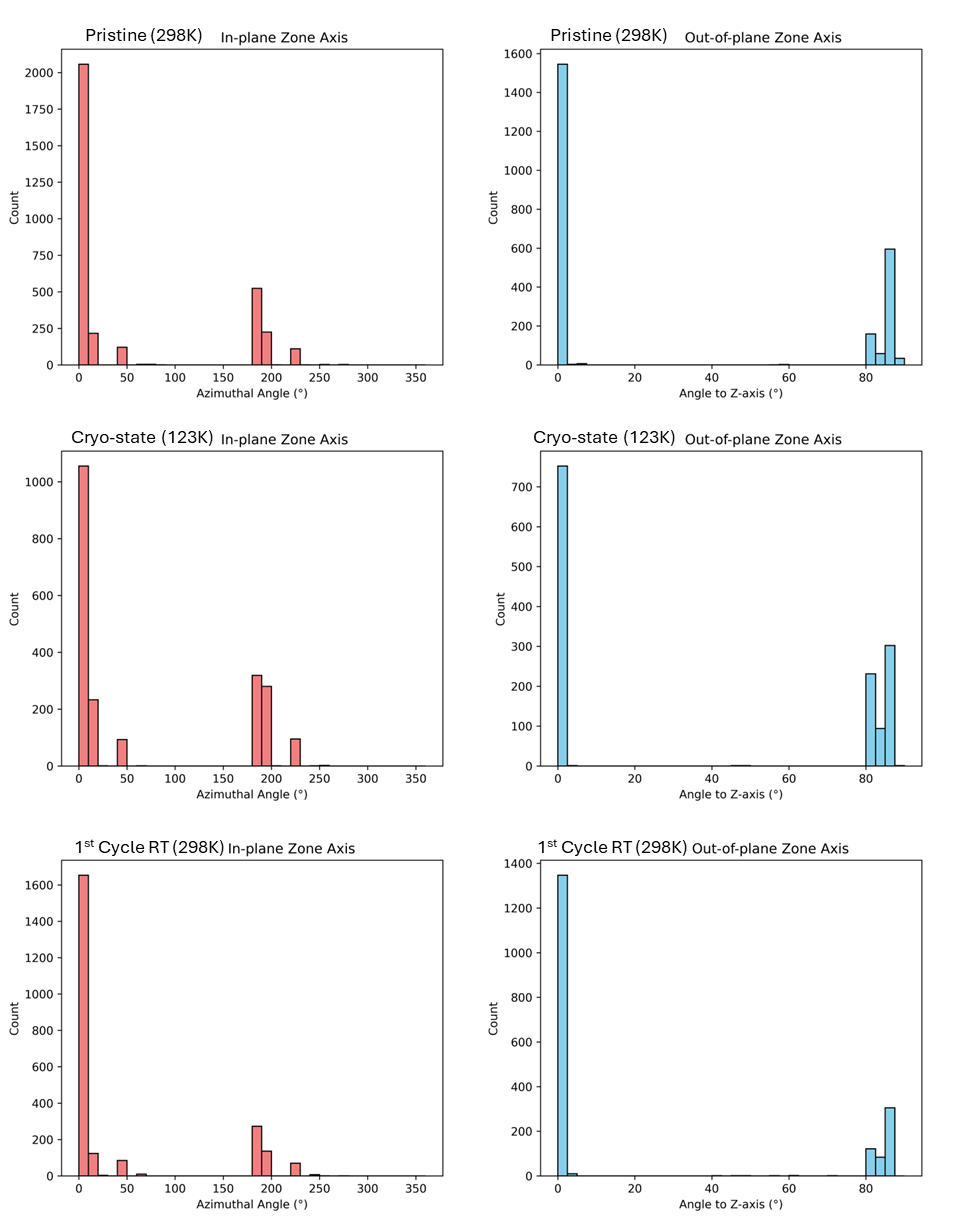


**Figure. S11.** The distribution of in-plane and out-of plane misorientation from its zone axis at pristine, cryo-state and 1^st^ cycle RT conditions


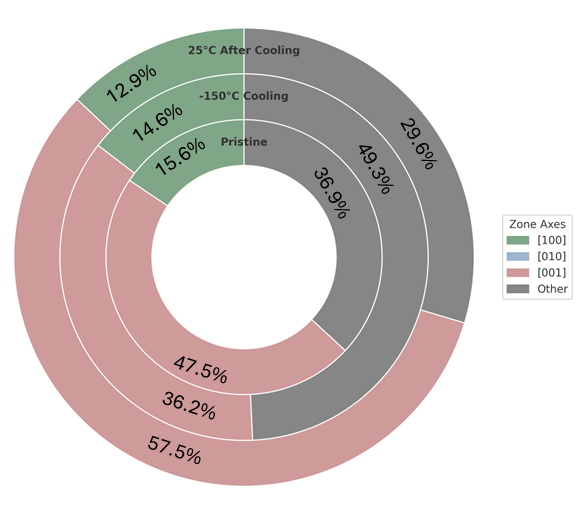


**Figure. S12.** The combined out-of plane zone axis distribution with 10 degrees tolerance for zone axes detection from Euler angle generated from orientation map for samples at pristine, cooling and reheated conditions.


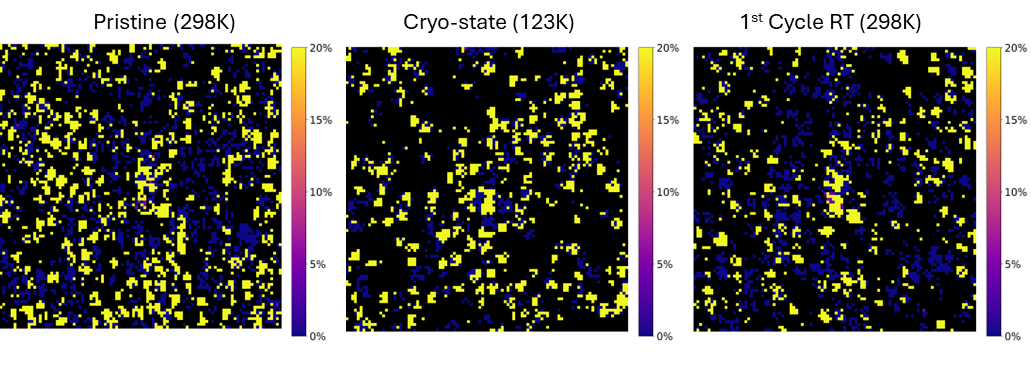


**Figure. S13.** Misorientation maps of tilt from [001] showing increased local tilting during cooling, associated with the formation of tilted monoclinic domains.


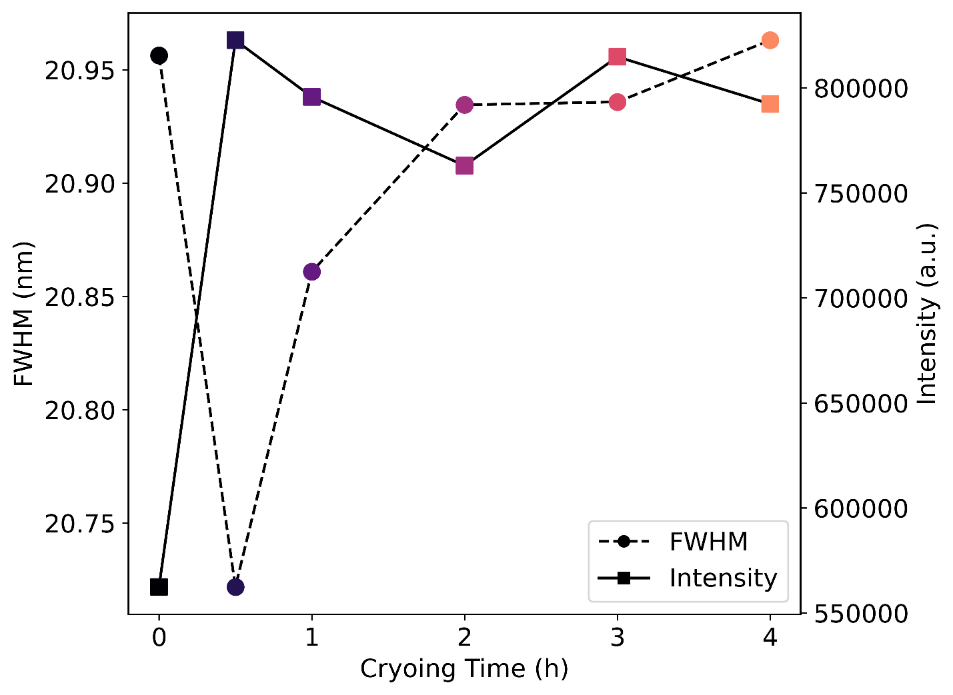


**Figure. S14.** Evolution of Gaussian-fitted FWHM and PL intensity of CsPbBr_3_ QDs after a single cryogenic cycle. The FWHM (dashed line with circular markers) and photoluminescence (PL) intensity (solid line with square markers) were extracted from PL spectra using Gaussian fitting. The quantum dots were cooled at 77 K for varying durations and then relaxed at room temperature for eight hours before measurement. This analysis reflects the optical response of CsPbBr_3_ QDs to different cryogenic exposure times within a single cooling cycle.


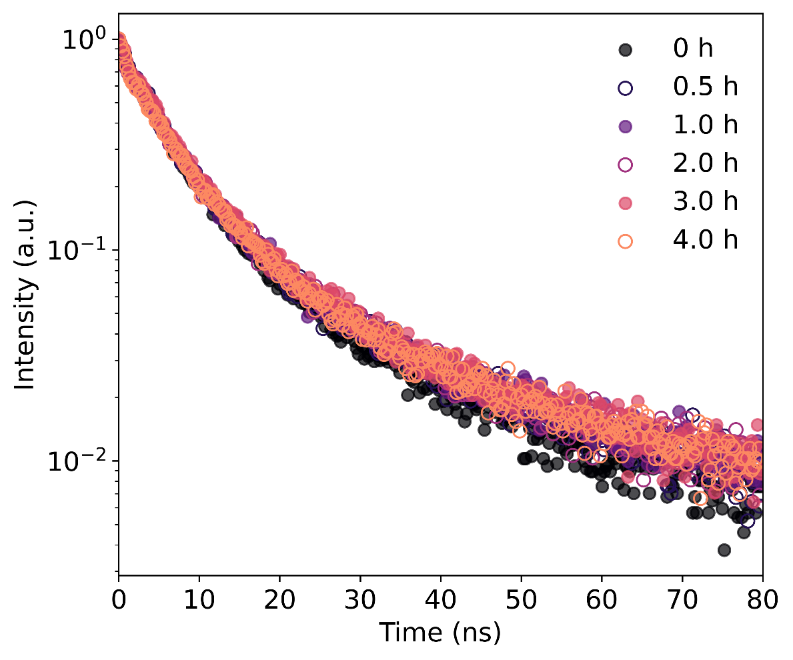


**Figure. S15.** Time-resolved photoluminescence (TRPL) measurements of CsPbBr_3_ QDs under varying cryogenic durations, revealing a corresponding increase in carrier lifetime during moderate freezing, followed by a lifetime reduction after extended cryogenic exposure.


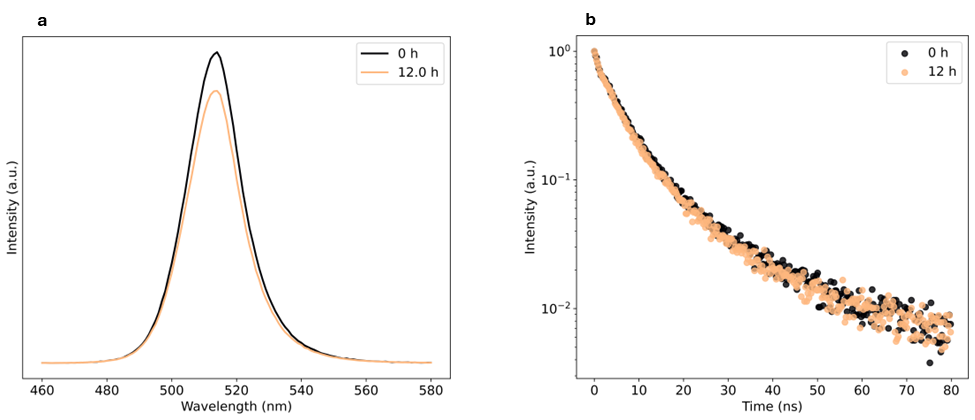


**Figure. S16.** Comparison of PL spectra and TCSPC decay profiles of CsPbBr_3_ QDs performed at 0 h (no cooling) and 12 h of cooling at 77 K. All samples were subsequently relaxed at room temperature for eight hours prior to measurement. The figure illustrates the effect of prolonged cryogenic treatment on the emission characteristics and carrier dynamics of the QDs.
